# Incidence and Predictors of Pacing‐Induced Cardiomyopathy in Paced Patients Undergoing Attempted Left Bundle Branch Area Pacing

**DOI:** 10.1111/jce.16767

**Published:** 2025-07-09

**Authors:** Katsuhide Hayashi, Aritra Paul, Roy Chung, Shady Nakhla, Chadi Tabaja, David O. Martin, Thomas Callahan, Bryan Baranowki, Mohamed Kanj, Tyler Taigen, Niraj Varma, Oussama Wazni, John Rickard

**Affiliations:** ^1^ Cardiac Electrophysiology and Pacing Section, Department of Cardiovascular Medicine Cleveland Clinic Cleveland Ohio USA; ^2^ Department of Heart Rhythm Management University of Occupational and Environmental Health, Japan Kitakyushu Japan; ^3^ Department of Cardiovascular Medicine Cleveland Clinic Abu Dhabi Abu Dhabi United Arab Emirates

**Keywords:** AVB, LBBAP, LVEF, PICM, V6RWPT

## Abstract

**Background:**

The incidence and predictors of pacing‐induced cardiomyopathy (PICM) in patients undergoing attempted left bundle branch area pacing (LBBAP) are unknown.

**Objective:**

To examine the incidence and predictors of PICM in patients with preserved left ventricular ejection fraction (LVEF) and atrioventricular block (AVB) undergoing attempted LBBAP.

**Methods:**

The study cohort included consecutive patients undergoing an attempt at LBBAP at the Cleveland Clinic from 2018 until 2022 with preserved LVEF and AVB. PICM was defined as post‐PM LVEF decrease to < 50% and > 5%, > 10% decrease from pre‐PM implantation. Patients who had alternative reasons for a decrease in LVEF during follow‐up were excluded. LBBAP was defined as meeting common criteria for LBBAP and the incidence of PICM was evaluated.

**Results:**

A total of 173 patients were included. PICM developed in 13/173 (7.5%) of patients at 26 (IQR 7–70) weeks after PM implantation. In total, 12 (6.9%) patients experienced LVEF > 10% decrease. Of 173 patients, 118 (68.2%) patients met criteria for LBBAP. The LBBAP group had a significantly lower incidence of PICM compared with the non‐LBBAP group (log rank *p* = 0.048). The optimal V6RWPT for predicting PICM was 80 ms and the incidence of PICM increased in proportion to the increase in V6RWPT. In multivariate analysis, non‐LBBA capture (HR: 5.58, 95% CI: 1.46–24.32; *p* = 0.01) and LVEF at pre‐PM implant per 10% (HR: 0.11, 95% CI: 0.02–0.40; *p* = 0.0003) were independent predictors for the development of PICM.

**Conclusion:**

The incidence of PICM with LBBAP in paced patients undergoing PM implant with AVB and preserved LVEF is low. LBBA capture was associated with freedom from PICM.

**Abbreviations**
AVB: atrioventricular block
DSP: deep septal pacing
HF: heart failure
LBBAP: left bundle branch area pacing
LVEF: left ventricular ejection fraction
PICM: pacing‐induced cardiomyopathy
RV: right ventricular
V6RWPT: time from pacing spike to R‐wave peak in leads V6

## 1 Introduction

Right ventricular (RV) pacing can lead to progressive left ventricular (LV) systolic dysfunction through electronic and mechanical inter‐ and intraventricular dyssynchrony [1]. This deleterious effect is termed pacing‐induced cardiomyopathy (PICM) [2, 3, 4]. PICM is not uncommon especially in atrioventricular (AV) block requiring significant amounts of RV pacing. In a large cohort study, Zhang and colleagues reported that 26% of chronically RV paced patients with high‐grade AV block and a normal left ventricular ejection fraction (LVEF) developed new clinical heart failure (HF) [5]. Previous reports have shown that percent RV pacing and a wider paced QRS complex are predictors for the development of PICM in patients requiring frequent RV pacing [5, 6, 7]. Left bundle branch area pacing (LBBAP) has emerged as an alternative method for physiological pacing [8, 9]. LBBAP is a term which encompasses direct LBB capture, fascicular capture, or LV septal capture. LV septal capture occurs when the pacing lead is in close proximity to the conduction system but does not directly capture it. The term deep septal pacing has emerged which refers to pacing relatively deep within the interventricular septum but not close to the LV conduction system. Physiological pacing produces favorable LV electronic and mechanical synchronous pacing [10]. As such, LBBAP may confer a lower risk for the development of PICM. However, LBBAP is not always possible due to anatomic issues resulting in deep septal pacing. In the current study, we sought to examine the incidence and predictors of PICM in patients with a preserved LVEF and AV nodal disease requiring a high percentage of ventricular pacing undergoing attempted LBBAP to assess whether achievement of LBBAP has an impact on the development of PICM compared to deep septal pacing.

## 2 Methods

### 2.1 Study Cohort

The cohort included consecutive patients undergoing an attempt at LBBAP with AV block and a preserved LVEF using the Medtronic Model 3830 lead (Select secure system, Medtronic Inc., Minneapolis, MN) at the Cleveland Clinic main campus and satellites between July 2018 and December 2022. All study patients were followed up by remote monitoring and regular outpatient visits.

Patients were excluded if they had documented failure to achieve even a deep septal position for the RV lead, had no preoperative or follow‐up transthoracic echocardiography, had infrequent ventricular pacing, or had an LVEF < 50% on preoperative echocardiography. Finally, patients who had a clear alternative reason for a decrease in LVEF during follow‐up were excluded.

Demographic, historical, echocardiographic, and electrocardiographic data, PM indications, PM programming settings at implantation, and pacing data using stored remote monitoring were obtained from the electronic medical record via retrospective chart review. Percent right atrial (RA), and RV pacing was recorded at the end of follow‐up. The study was approved by the Institutional Review Board of the Cleveland Clinic.

#### 2.1.1 Procedure Description

The LBBAP implantation procedure was described in detail elsewhere [8, 11]. All the procedures were performed using a fixed curve or deflectable sheath (Medtronic C315 His sheath or C304 His sheath), and the 4.1F, active helix, screw‐in pacing lead (Model 3830; Medtronic) screwed deep into the septum with the goal of left conduction system capture. Total operators were 9 physicians, and all physicians had > 10 years' experience as cardiac device implanter. In terms of LBBAP implantation, procedure experience grew during the study period as it includes data from 2018 when LBBAP first emerged.

#### 2.1.2 Electrocardiographic Definition and Measurements

In our study, left bundle branch area capture was defined based on any of the following criteria [12].

1. QRS morphology transition during threshold test [8, 13].
2. QRS morphology transition during programmed stimulation [14].
3. Pacing stimulus to V6RWPT < 80 ms in patients with a narrow QRS/isolated right branch block patients or < 90 ms in patients with more advanced ventricular conduction system disease [13, 15].

The first two criteria were assessed during chart review. The third was prospectively measured. Electrocardiographic measurements were performed using the paced 12‐lead ECG magnified 5 times using digital calipers at 25 mm/s sweep speed using the MUSE Cardiology information system (GE HealthCare Technologies Inc., Chicago, IL). In each patient, the paced QRS duration and the time from pacing spike to R‐wave peak in leads V6 (V6RWPT) were carefully measured.

#### 2.1.3 Outcomes

PICM was defined as (1) both a post‐PM LVEF decrease to < 50% and > 5% decrease and (2) both a post‐PM LVEF decrease to < 50% and > 10% decrease from pre‐PM implantation via echocardiography. Time to outcome was calculated from the date of PM implantation to the follow‐up echocardiogram.

#### 2.1.4 Statistical Analysis

Continuous variables were expressed as mean ± standard deviation (SD) or median with interquartile range, IQR: 25th–75th depending on the distribution of data. Categorical variables were expressed as counts and percentages. Student's *t*‐test, *χ* ^2^ test, or Fisher's exact test were used to compare continuous and categorical variables. The Kaplan–Meier method and Cox proportional hazard regression analysis were used to identify the clinical predictors of the development of PICM. Gender, LVEF at pre‐PM implant, paced QRS duration, and non‐LBB area capture based on prior knowledge were entered into a multiple variable regression model looking at the prediction of PICM. *p* < 0.05 was considered statistically significant. All statistical analyses were performed using JMP version 17.0.0 (SAS Institute Inc., Cary, NC, USA).

## 3 Results

### 3.1 Subjects Demographics

**Figure 1:**
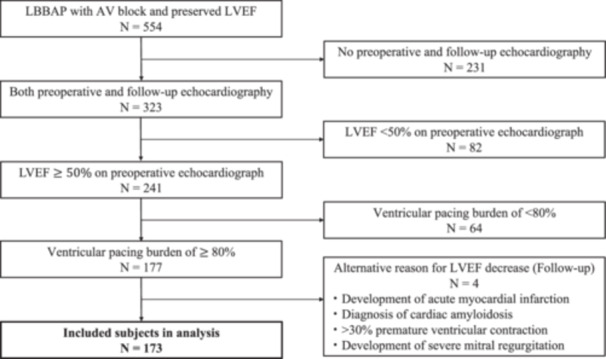
Patient flow chart.

A total of 565 consecutive patients were screened. A patient flow chart is shown in Figure 1. Patients who met the exclusion criteria were excluded as follows: 11 patients had an LBB area pacing (LBBAP) attempt that was abandoned in favor of RV septal pacing where no attempt was made to screw the lead deep into the septum. Other reasons for exclusion included: no preoperative and follow‐up transthoracic echocardiography (*n* = 231), ventricular pacing burden of < 80% following PM implantation (*n* = 64), and an LVEF < 50% on preoperative echocardiography (*n* = 82). Finally, patients who had a clear alternative reason for a decrease in LVEF during follow‐up (developing an acute myocardial infarction, new diagnosis of cardiac amyloidosis, development of a > 30% premature ventricular contraction burden, and development of severe mitral regurgitation (*n* = 4)) were excluded. The remaining 173 patients were included in the study.

Baseline patient characteristics are listed in Table 1. The mean patient age was 72 ± 12 years. A total of 105 patients (60.7%) were male. More than 50% of patients had atrial arrhythmias and one‐third of patients had ischemic heart disease. The mean LVEF at pre‐PM implantation was 60 ± 6%. The paced QRS duration was 136 ± 19 ms and the V6RWPT was 80 (IQR, 70–90) ms, respectively.

**Table 1:** Baseline patient characteristics (*N* = 173).

Demographics	
Age (years)	72 ± 12
Gender: male, *n* (%)	105 (60.7)
Medical history, [*n* (%)]	
Atrial arrhythmia	99 (57.3)
Hypertension	118 (68.2)
Diabetes mellitus	42 (24.3)
Ischemic heart disease	60 (34.7)
COPD	19 (11.0)
ESRD on hemodialysis	0 (0)
Echocardiographic measurements	
Left ventricular ejection fraction, %	60 ± 6
Intrinsic QRS type	
Narrow QRS	27 (15.6)
RBBB	21 (12.1)
RBBB + LAFB/LPFB	17 (9.8)
NIVCD	38 (22.0)
LBBB	17 (9.8)
Alternating bundle branch block	1 (0.6)
Escape with narrow QRS (junctional rhythm)	5 (2.9)
Escape with RBBB‐type QRS	20 (11.6)
Escape with LBBB‐type QRS	13 (7.5)
Complete asystole (paced)	12 (6.9)
Uncertain	2 (1.2)
Paced QRS duration, ms	136 ± 19
V6/V1 interpeak interval, ms	28 (16–40)
V6RWPT, ms	80 (70–90)
Intrinsic narrow QRS or RBBB morphology	78 (69–88)
Intrinsic wide QRS or LBBB morphology	83 (73–92)

*Note:* Continuous variables were expressed as mean ± standard deviation (SD) or median (IQR: 25%–75%); categorical data are presented as number (percentage).

Abbreviations: COPD = chronic obstructive pulmonary disease, ESRD = end‐stage renal disease; LAFB = left anterior fascicular block, LAFB = left posterior fascicular block, LBBB = left bundle branch block, NIVCD = nonspecific intraventricular conduction disturbance, RBBB = right bundle branch block, V6RWPT = time from pacing spike to R‐wave peak in leads V6.

### 3.2 Pacemaker Programming and Interrogation Data

Pacemaker programming at the time of implantation is shown in Table 2. DDD mode was the most common and rate‐responsive pacing was programmed in 45% of patients. The lower rate limit (LRL) was programmed to 62 ± 8 bpm. Bipolar ventricular pacing polarity was programmed in 82% of the patients. The cumulative percent of RA and RV pacing (%RA pacing and %RV pacing) was a median of 16 (IQR, 0%–56%) and 99 (IQR, 80%–100%), respectively.

**Table 2:** Pacemaker programming and interrogation data.

Pacing mode	
DDD	91 (52.6)
DDDR	62 (35.8)
DDI	1 (0.6)
DDIR	4 (2.3)
VVI	2 (1.2)
VVIR	13 (7.5)
Pacing rate, ppm	
Lower rate limit	62 ± 8
Max tracking rate limit	129 ± 10
Atrioventricular interval, ms	
Sensed	148 ± 29
Paced	177 ± 26
Ventricular pacing	
Unipolar	32 (18.5)
Bipolar	141 (81.5)
Cumulative% of	
Atrial paced	16 (0–56)
Ventricular paced	99 (80–100)

*Note:* Continuous variables were expressed as mean ± standard deviation (SD) or median (IQR: 25%–75%); categorical data are presented as number (percentage). DDD, DDI, VVI, non‐rate responsive pacing; DDDR, DDIR, VVIR, rate responsive pacing.

### 3.3 Outcome

**Figure 2:**
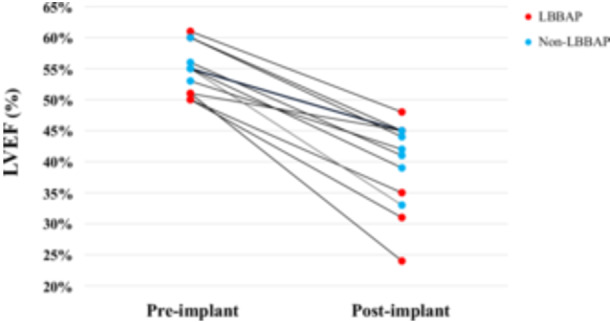
LVEF change from pre‐ to postimplant.

**Figure 3:**
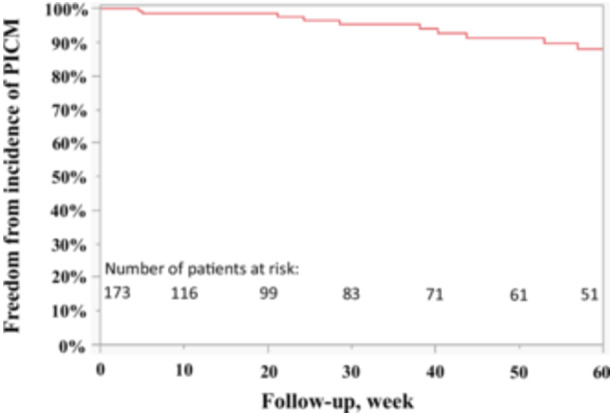
Survival‐free rate from PICM in all patients.

**Figure 4:**
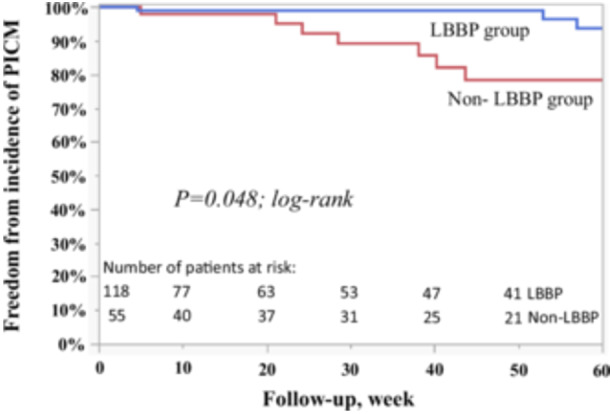
Survival‐free rate from PICM by LBBP versus non‐LBBP group.

In total, 13/173 (7.5%) of patients experienced the endpoint of an LVEF decrease to < 50% and > 5% decrease from pre‐PM implantation at 26 (IQR 7–70) weeks after PM implantation and 12/173 patients (6.9%) experienced an LVEF decrease to < 50% and > 10% decrease from preimplant. Of the 13 patients who met the primary endpoint, 2 patients (15.4%) were hospitalized for HF and no patients were upgraded to CRT during follow‐up. Detailed information and the clinical course of the 13 patients who developed PICM are summarized in Table 3. The LVEF change from pre‐PM implantation to post‐PM implantation is shown in Figure 2. Of 173 patients, 118 (68.2%) patients met criteria for LBBAP. Table 4 summarizes a comparison of patient characteristics and procedural outcomes between the LBBAP and non‐LBBAP groups. The LBBAP group experienced a 4.2% (5 of 118) incidence of PICM whereas the non‐LBBAP group experienced a 14.6% (8 of 55) incidence of PICM. A similar trend was observed when PICM was defined as post‐PM LVEF decrease to < 50% and > 10% decrease (the LBBAP group experienced a 4.2% (5 of 118) incidence of PICM whereas the non‐LBBAP group experienced a 12.7% (7 of 55) incidence of PICM, *p* = 0.054). Figure 3 shows the survival‐free rate from PICM in all patients. The LBBAP group had a significantly lower incidence of PICM versus those in the non‐LBBAP group (Figure 4).

**Table 3:** Detailed information and clinical course of 13 patients developed PICM.

	LBBAP	Pre‐LVEF, %	Post‐LVEF, %	V6RWPT, ms	Paced QRS duration, ms	CRT upgrade	HF hospitalization
No. 1	No	56	41	100	150	No	No
No. 2	No	50	35	91	144	No	No
No. 3	No	51	24	85	144	No	No
No. 4	Yes	50	31	80	156	No	Yes
No. 5	Yes	60	45	65	123	No	No
No. 6	Yes	61	48	80	155	No	No
No. 7	No	55	45	120	184	No	No
No. 8	No	51	45	104	162	No	Yes
No. 9	Yes	55	33	89	136	No	No
No. 10	No	55	39	98	152	No	No
No. 11	No	55	45	100	136	No	No
No. 12	Yes	60	44	80	113	No	No
No. 13	No	53	42	93	112	No	No

Abbreviations: CRT = cardiac resynchronization therapy; HF = heart failure; LBBP = left bundle branch pacing; LVEF = left ventricular ejection fraction; PICM = pacing‐induced cardiomyopathy; V6RWPT = time from pacing spike to R‐wave peak in leads V6.

**Table 4:** Comparison of patient characteristics and procedural outcome between the LBBAP group and the non‐LBBAP group.

	LBBAP group *n* = 118	Non‐LBBAP group *n* = 55	*p*
Demographics			
Age, year	72 ± 11	72 ± 13	0.81
Gender: male, *n* (%)	66 (55.9)	39 (70.9)	0.07
Medical history: *n* (%)			
Hypertension	76 (64.1)	42 (76.4)	0.16
Diabetes mellitus	32 (27.1)	10 (18.2)	0.25
Ischemic heart disease	38 (32.2)	22 (40.0)	0.39
COPD	15 (12.7)	4 (7.3)	0.43
LVEF at preimplant, %	60 ± 6	61 ± 7	0.4
Intrinsic wide QRS or LBBB morphology, *n* (%)	71 (60.2)	27 (49.1)	0.19
Bipolar at ventricular pacing polarity, *n* (%)	96 (81.4)	45 (81.8)	1
ECG measurements			
V6RWPT, ms	75 (66–80)	94 (99–100)	< 0.0001
Paced QRS duration, ms	132 ± 19	143 ± 19	0.0005
V6/V1 interpeak interval, ms	32 (20–44)	22 (12–28)	< 0.0001
Procedural outcome			
Fluoroscopy time, min	12 (8–19)	14 (8–23)	0.27
Total procedure time, min	98 (78–120)	104 (83–130)	0.18
Complication, *n* (%)	0	0	1

*Note:* Continuous variables were expressed as mean ± standard deviation (SD) or median (IQR: 25%–75%); categorical data are presented as number (percentage).

Abbreviations: COPD = chronic obstructive pulmonary disease, ECG = electrocardiography, LBBB = left bundle branch block, LVEF = left ventricular ejection fraction, V6RWPT = time from pacing spike to R‐wave peak in leads V6.

### 3.4 Association of Paced QRS Duration, V6RWPT and Development of PICM

**Figure 5:**
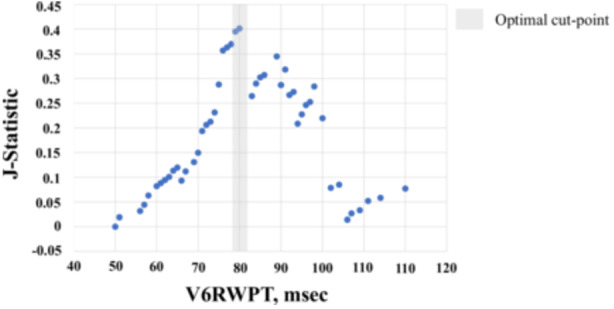
Optimal V6RWPT for predicting PICM.

There was a nonsignificant trend toward patients developing PICM having a wider paced QRS duration than those without PICM (144 ± 20 vs. 135 ± 19, *p* = 0.12) and the incidence of PICM tended to be higher in patients with a paced QRS duration > 150 ms compared with the patients with paced QRS duration ≤ 150 ms (5 of 35, 14.3% vs. 8 of 138, 5.8%, *p* = 0.14). Two of 13 (15.4%) patients who met the criteria for LBBAP with a paced QRS duration > 150 ms developed PICM. Patients who developed PICM had a significantly longer V6RWPT compared to those who maintained a normal LVEF (91 ± 14 vs. 80 ± 14 ms, *p* = 0.007). Table 5 summarizes ECG measurements between the patients with and without the development of PICM. The optimal V6RWPT for predicting PICM was chosen to be 80 ms based on *J*‐statistics (Figure 5).

**Table 5:** ECG measurements between the patients developed or nondeveloped PICM.

	Developed PICM (*N* = 13)	Nondeveloped PICM (*N* = 160)	*p*
R‐wave in V1, %	7 (53.9)	98 (61.3)	0.77
Paced QRS duration, ms	144 ± 20	135 ± 19	0.12
V6RWPT, ms	80 ± 14	91 ± 14	0.007
V6/V1 interpeak distance, ms	26 ± 15	29 ± 15	0.50

*Note:* Continuous variables were expressed as mean ± standard deviation (SD; categorical data are presented as number (percentage).

Abbreviations: PICM = pacing‐induced cardiomyopathy, V6RWPT = time from pacing spike to R‐wave peak in leads V6.

Table 6 shows the comparison of results between the three groups classified by V6RWPT. The incidence of PICM increased in proportion to the increase in V6RWPT (1 of 77, 1.3% in the patients with V6RWPT < 80 ms vs. 5 of 48, 10.4% in V6RWPT 80–89 ms vs. 7 of 48, 14.6% in V6RWPT ≥ 90 ms, *p* = 0.02). There was no difference in the population of patients who were set at bipolar pacing between the three groups. Furthermore, the percentage of paced R wave morphology in V1 was greater in patients with V6RWPT < 80 ms (79%) compared to 56% in patients with V6RWPT 80‐89 ms and 35% in V6RWPT ≥ 90 ms (*p* < 0.0001). In addition, paced QRS duration increased in proportion to increase in V6RWPT (*p* < 0.0001).

**Table 6:** Comparison between three groups by V6RWPT.

	V6RWPT < 80 ms (*N* = 77)	V6RWPT 80–90 ms (*N* = 48)	V6RWPT ≥ 90 ms (*N* = 48)	*p*
LVEF at baseline, %	60 ± 6	60 ± 6	60 ± 7	0.95
Bipolar pacing	66 (86)	39 (81)	36 (75)	0.32
R‐wave in V1	61 (79)	27 (56)	17 (35)	< 0.0001
V6RWPT, ms	68 ± 8	83 ± 3	99 ± 8	< 0.0001
Paced QRS duration, ms	127 ± 17	138 ± 16	147 ± 20	< 0.0001
Incidence of PICM	1/77 (1.3)	5/48 (10.4)	7/48 (14.6)	0.02

*Note:* Continuous variables were expressed as mean ± standard deviation (SD; categorical data are presented as number (percentage).

Abbreviations: LVEF = left ventricular ejection fraction, PICM = pacing‐induced cardiomyopathy, V6RWPT = time from pacing spike to R‐wave peak in leads V6.

### 3.5 Predictors for Incidence of PICM

In multivariable analysis, LVEF at pre‐PM implant per 10% (HR: 0.11, 95% CI: 0.02–0.40; *p* = 0.0003) and non‐LBB area capture (HR: 5.58, 95% CI: 1.46–24.32; *p* = 0.01) were independent predictors for the development of PICM (Table 7).

**Table 7:** Predictors for PICM.

	Univariate analysis		Multivariable analysis	
	HR (95% CI)	*p*	HR (95% CI)	*p*
Age, per 1 year	0.99 (0.95–1.04)	0.79		
Gender, male	0.53 (0.17–1.64)	0.27	0.36 (0.09–1.31)	0.12
Atrial arrhythmias	1.75 (0.52–5.92)	0.37		
Coronary artery disease	0.54 (0.14–2.05)	0.37		
Hypertension	1.60 (0.42–6.08)	0.49		
Diabetes mellitus	0.93 (0.24–3.55)	0.92		
Left ventricular ejection fraction at pre‐implant, 10%	0.11 (0.02–0.40)	0.0003	0.11 (0.02–0.40)	0.0003
Paced QRS duration, 10 ms	1.26 (0.78–0.94)	0.12	1.05 (0.73–1.51)	0.78
Non‐LBB area capture (vs. LBB area capture)	3.85 (1.22–13.30)	0.02	5.58 (1.46–24.32)	0.01
Unipolar at ventricular pacing polarity	2.10 (0.60–7.29)	0.27		
Terminal r in V1	0.74 (0.24–2.30)	0.6		
Intrinsic LBBB/NICD/wide escape/asystole morphology	1.63 (0.53–5.06)	0.4		
Rate response on	0.57 (0.17–1.93)	0.37		
Lower rate limit, per 1 ppm	0.99 (0.93–1.07)	0.93		
%RA pacing, 10%	1.08 (0.92–1.28)	0.36		
%RV pacing, 10%	1.05 (0.87–1.26)	0.63		

Abbreviations: CI = confidence interval, HR = hazard ratio, LBBB = left bundle branch; NIVCD = nonspecific intraventricular conduction disturbance, PICM = pacing‐induced cardiomyopathy, RA = right atrium, RV = right ventricle, V6RWPT = time from pacing spike to R‐wave peak in leads V6.

## 4 Discussion

To the best of our knowledge, this report is the first to investigate the incidence of PICM by LBBAP in ventricular paced patients with AV block and a preserved LVEF. In the current study, we noted several important findings. First, we found that the patients who achieved left bundle area pacing had a significantly lower incidence of PICM compared to those who did not achieve LBBAP. Second, the incidence of PICM increased in proportion to the increase in V6RWPT such that patients who achieve a V6RWPT < 80 ms had only a 1.3% risk of PICM. Finally, our results indicate that non‐LBB area capture was an independent predictor for the development of PICM. These data suggest that conduction system engagement is important to prevent the development of PICM in patients with a preserved LVEF and AV block.

### 4.1 Incidence of Development of PICM by LBBAP in Patients With AV Block and Preserved LVEF

In this study of paced patients undergoing attempted LBBAP with AV block and a preserved LVEF, 7.5% developed PICM at 26 (IQR 7–70) weeks after PM implantation. The definition of PICM varies between studies, but our definition (post‐PM LVEF decrease to < 50% and > 5% decrease from pre‐PM implantation) used in this study can be interpreted broadly. The incidence of PICM in patients with a preserved LVEF and apical RV pacing has been reported to be up to 26% during 4.8 ± 2.2 years of follow‐up [5, 6, 7, 16]. Of interest is whether attempting LBBAP is better than RV apical pacing. Based on historical incidences of PICM, this finding cannot be confirmed due to the differences in follow‐up. However, our results suggest that the patients who achieved left bundle branch area capture had a significantly lower incidence of PICM compared to those who did not achieve left bundle branch area capture.

LBBAP produces favorable LV electrical and mechanical synchronous activation which likely explains the current results. In a recent meta‐analysis of conduction system pacing, the authors showed that conduction system pacing significantly improved cardiac function, promoted reverse ventricular remodeling, and provided stable electrical parameters for patients who already developed a PICM by RV pacing [17]. In this study, HB pacing was not widely performed due to its operative difficulty and increased capture thresholds. LBBAP offers a more practical way of mitigating dyssynchrony and is thought to activate the left‐sided conduction system similarly to HB pacing [18, 19, 20]. The hemodynamic changes associated with deep septal pacing are largely unknown. In this study, the incidence of the development of PICM in the LBBAP group was non‐zero. There are several possible reasons. First, this may be due to occur by a non‐PICM etiology. We confirmed all potential alternative etiologies of decline in LVEF and excluded the eligible patients from analysis as possible. However, not all the patients underwent detailed examination for cardiomyopathy, etc. Second, there may be a physiologic basis for development of PICM even if the conduction system is being engaged. The incidence of PICM tended to be higher in patients with a paced QRS duration > 150 ms even if patients met the criteria for LBBAP. The fact, however, remains that the rates for development of PICM were non‐zero even in patients with a shorter V6RWPT, r/R′ in V1, and a narrow QRS duration by LBBAP. Whether this represents a true pacing cardiomyopathy or simply the development of another process not easily identified is unclear. Upgrade of these patients to biventricular pacing with the addition of a coronary sinus lead could have shed some light on this however was not performed in the current cohort.

### 4.2 Achievement of LBBAP and the Risk for Development of PICM

LBBAP is defined as the capture of the subendocardial area on the left side of the interventricular septum which includes left bundle branch pacing (LBBP), left fascicular pacing (LFP), and left ventricular septal pacing (LVSP) based on the European Heart Rhythm Association (EHRA) clinical consensus statement 2023 [21]. However, in reality, differentiation of LBBP/LFP/LVSP is very challenging. Therefore, we categorized the entire cohort into LBBA area pacing versus non‐LBBAP. In the current study, bipolar pacing may have affected the QRS morphology and have caused the disappearance of the R‐wave in V1 due to anodal capture of the subendocardial area on the right side of the interventricular septum. However, our results revealed that both the ventricular pacing polarity, whether unipolar or bipolar, and the presence or absence of an R‐wave in V1 did not impact the development of PICM. More importantly, pacing polarity should not affect the V6RWPT. Therefore, we reached the conclusion that the achievement of LBBAP and a shorter V6RWPT was associated with a lower incidence of PICM. Patients with DSP would be expected to have a wider V6RWPT due to increased transit times across the septum compared to LBBAP. While the lead position could affect V6RWPT, its effect is likely small. As such, based on this data, implanters should make every effort to achieve LBBAP when possible.

### 4.3 Predictors of PICM by LBBAP

Paced QRS duration, %RV pacing, and lower pre‐implant LVEF are well‐known risk factors for the development of PICM in patients with AV block [5, 6, 7, 16]. Our results revealed that even slightly lower pre‐implant LVEF measurements within the normal range appeared to be a predictor of PICM. In our study, with respect to V6RWPT, the *J*‐statistic revealed that the optimal cut point of V6RWPT for the development of PICM was 80 ms in this population (Figure 3). Interestingly, Jastrzębski and colleagues showed that a V6RWPT value < 74 ms had a specificity of 100% for LBB capture in patients who had an intrinsic narrow QRS or RBBB and < 80 ms in patients who had an intrinsic LBBB/NICD/wide escape/asystole in an experimental study [13]. Taking into consideration these results, coupled with the current study, it appears that LBB area capture is important in the prevention of PICM. We could not identify each optimal cut‐point of V6RWPT for patients who had either an intrinsic narrow QRS/RBBB and or who had an intrinsic LBBB/NICD/wide escape/asystole due to statistical underpowering.

While a wider paced QRS duration was not predictive for the development of PICM in the current study, there was a nonsignificant trend. This was likely due to statistical underpowering as there was a trend toward a wider paced QRS duration in patients who developed PICM compared to those who maintained a normal LVEF (144 ± 20 vs. 135 ± 19, *p* = 0.12). %RV pacing was not associated with the development of PICM in this population as almost all subjects were ventricular pacing dependent with a median value of %RV pacing of 99% (IQR, 80%–100%).

## 5 Study Limitations

Our study has several notable limitations. This was a single‐center retrospective analysis and, therefore, may be affected by unknown confounders. Multicenter experiences are warranted. It is possible that in patients where non‐LBBAP was settled on could represent a sicker cohort of patients and that the patients in non‐LBBAP arm could have had subtly different substrates which may have made LBB capture more difficult, resulting in worse outcomes in the non‐LBBAP group. However, given that the ejection fraction in all patients was preserved at baseline makes this somewhat less likely. In addition, we ran the analysis with other criteria for LBB pacing using a V6RWPT < 74 ms (non‐LBBB) or < 80 ms (LBBB) [22]. In these criteria, the LBBAP group experienced a 2.9% (2 of 70) incidence of PICM whereas the non‐LBBAP group experienced a 10.7% (11 of 103) incidence of PICM (*p* = 0.08). Relatively short‐term follow‐up in this study may have underestimated the true PICM incidence. The population was not large enough to determine the relationship between paced QRS duration and the development of PICM. Regarding the measurements of V6/V1 interpeak differences, more than 80% of study patients were programmed to bipolar pacing which might result in anodal capture and impact that number. We could not distinguish in detail the LBB capture site. This may have had some effect on V6RWPT. That said, based on our definitions, we are confident that we could reasonably distinguish between LBBAP and deep septal pacing. LBBAP, by stylet‐driven leads at the Cleveland Clinic, did not come into practice until very recently. As such, if the current findings can be extrapolated to stylet‐driven leads is unclear. We could also not determine the electrode depth in the ventricular septum by imaging tools, as this was a retrospective study, and follow‐up echocardiography was not performed specifically to look at this measurement. We did not obtain ECGs in both a VVI setup and a DDD setup to evaluate fusion in our routine device follow‐up. Therefore, the effect of fusion could not be determined. Lastly, we had no systematic echocardiography follow‐up protocol and hence many patients were excluded due to not having pre‐ and follow‐up echocardiograms, therefore, this may have affected the incidence of PICM.

## 6 Conclusion

The incidence of PICM in patients who required permanent pacing with a preserved LVEF and significant AV nodal disease undergoing an attempt at LBBAP was 7.5% at 26 weeks after PM implantation. Achievement of LBBAP had a significant impact on reducing the development of PICM compared with deep septal pacing. In patients who had a V6RWPT < 80 ms, only 1.3% developed PICM. These data suggest that physicians should make a concerted effort to achieve LBBAP targeting a V6RWPT < 80 ms and to avoid DSP when possible.

## Conflicts of Interest

Katsuhide Hayashi, Aritra Paul, and Chadi Tabaja—nothing to disclosure. Roy Chung and David O. Martin—consulting: Medtronic. Shady Nakhla—research grants from Philips. Tomas Callahan—consulting: Philips, Medtronic, Abbott, Boston Scientific, Shockwave. Bryan Baranowski—teaching and speaking: Biosense Webster. Mohamed Kanji—teaching and speaking: Boston Scientific. Tyler Taigen—consulting: Biosense Webster, Medtronic. Niraj Varma—research/consulting: Abbott, Biotronik, Boston Scientific, EP solutions, Implicity, Impulse Dynamics, Medtronic, Pacemate. Oussama Wazni—consulting: Medtronic, Biosense Webster, Boston Scientific. John Rickard—research grants from Abbott; speaking: Boston Scientific.

## Data Availability

The data underlying this article will be shared on reasonable request to the corresponding authors.
